# Chronic treatment of curcumin improves hepatic lipid metabolism and alleviates the renal damage in adenine-induced chronic kidney disease in Sprague-Dawley rats

**DOI:** 10.1186/s12882-019-1621-6

**Published:** 2019-11-21

**Authors:** Hardik Ghelani, Valentina Razmovski-Naumovski, Dennis Chang, Srinivas Nammi

**Affiliations:** 1 School of Science and Health, Western Sydney University, Sydney, NSW 2751 Australia; 2 NICM Health Research Institute, Western Sydney University, Sydney, NSW 2751 Australia; 3 South Western Sydney Clinical School School of Medicine, University of New South Wales, Sydney, NSW 2052 Australia

**Keywords:** Curcumin, Chronic kidney disease, Dyslipidaemia, Adenine-induced chronic kidney disease

## Abstract

**Background:**

Chronic kidney disease (CKD), including nephrotic syndrome, is a major cause of cardiovascular morbidity and mortality. The literature indicates that CKD is associated with profound lipid disorders due to the dysregulation of lipoprotein metabolism which progresses kidney disease. The objective of this study is to evaluate the protective effects of curcumin on dyslipidaemia associated with adenine-induced chronic kidney disease in rats.

**Methods:**

Male SD rats (*n* = 29) were divided into 5 groups for 24 days: normal control (*n* = 5, normal diet), CKD control (*n* = 6, 0.75% w/w adenine-supplemented diet), CUR 50 (*n* = 6, 50 mg/kg/day curcumin + 0.75% w/w adenine-supplemented diet), CUR 100 (*n* = 6, 100 mg/kg/day curcumin + 0.75% w/w adenine-supplemented diet), and CUR 150 (*n* = 6, 150 mg/kg/day curcumin + 0.75% w/w adenine-supplemented diet). The serum and tissue lipid profile, as well as the kidney function test, were measured using commercial diagnostic kits.

**Results:**

The marked rise in total cholesterol, low-density lipoprotein (LDL) cholesterol, very low-density lipoprotein (VLDL) cholesterol, triglycerides and free fatty acids in serum, as well as hepatic cholesterol, triglyceride and free fatty acids of CKD control rats were significantly protected by curcumin co-treatment (at the dose of 50, 100 and 150 mg/kg). Furthermore, curcumin significantly increased the serum high-density lipoprotein (HDL) cholesterol compared to the CKD control rats but did not attenuate the CKD-induced weight retardation. Mathematical computational analysis revealed that curcumin significantly reduced indicators for the risk of atherosclerotic lesions (atherogenic index) and coronary atherogenesis (coronary risk index). In addition, curcumin improved kidney function as shown by the reduction in proteinuria and improvement in creatinine clearance.

**Conclusion:**

The results provide new scientific evidence for the use of curcumin in CKD-associated dyslipidaemia and substantiates the traditional use of curcumin in preventing kidney damage.

## Background

Chronic kidney disease (CKD) encompasses a spectrum of pathophysiological processes associated with abnormal kidney function (such as proteinuria) and a progressive decline in glomerular filtration rate. CKD is a substantial health problem, and its prevalence is increasing worldwide at least in part, due to a rise in the prevalence of systemic diseases such as metabolic syndrome that damage the kidney function [1, 2].

It is well documented that cardiovascular disease, such as heart failure or coronary artery disease, is one of the leading causes of mortality in patients with CKD. Thus, most patients with CKD die of the cardiovascular disease before dialysis becomes necessary [3–5]. CKD is associated with cardiovascular complications such as dyslipidaemia, atherosclerosis and myocardial infarction [6]. Patients and experimental animals with CKD have a high plasma concentration of lipid markers such as cholesterol, tryglycerides and fatty acids [7, 8].

The current therapeutic regimens including the use of statins and fibrates have limited success in treating the associated dyslipidaemia of CKD and do not address the underlying causal factors [9]. Although statins can be effective in slowing CKD progression in patients with mild-to-moderate CKD, they have consistently failed to alleviate HDL deficiency [9, 10]. Fibrates are indicated when hypertriglyceridaemia is the primary lipid abnormality in the CKD patient and may reduce triglyceride levels significantly [10]. However, fibrates are excreted by the kidney and may cause myositis, particularly when used in conjunction with statins [11]. Therefore, the development of novel therapies to either slow or reverse the deterioration in kidney function, as well as ameliorate the metabolic dyslipidaemia of CKD, is highly needed. Natural products have shown significant potential in improving hepatic lipid metabolism in experimentally-induced CKD [12–15].

Curcumin [1,7-bis (4-hydroxy-3-methoxyphenyl)-1,6-heptadiene-3,5-dione] is produced in the rhizome of the plant *Curcuma longa* L. and is a major polyphenolic chemical component of turmeric powder [16]. The pharmacokinetic, pharmacodynamics and clinical pharmacological properties of curcumin have been extensively studied over the past six decades. The effects of curcumin on renal damage have been investigated both in vivo and in vitro. In laboratory animals, chronic supplementation of curcumin has been shown to protect renal damage in various chemically-induced nephrotoxicity and renal injury models [17–29]. Curcumin has also been shown to possess renoprotective effects against various metal-induced nephrotoxicity [30–33]. In addition, the daily administration of curcumin has been shown to reduce proteinuria, glomerulosclerosis, tubule-interstitial injury and subsequently, renal failure in 5/6 nephrectomised rats [34–38]. Furthermore, curcumin has been shown to protect renal damage in streptozotocin- (STZ) [39–41], STZ-nicotinamide [42] and ischaemia-reperfusion-induced of CKD rat models [43]. In addition, curcumin has shown cardioprotective effects by attenuating chronic renal failure-induced cardiac hypertrophy and remodelling in 5/6 nephrectomised rats [44–46]. Recently, Abeer and El-Mahalaway [47] demonstrated that the daily administration of curcumin protected against aflatoxin-induced renal cortical damage in Wistar rats. Jacob et al. [48] observed that curcumin administration reduced glomerulonephritis injury inflammation and fibrosis and improved renal function in mice. In an in vitro study performed by Waly et al. [49], curcumin markedly protected the human embryonic kidney cell from cisplatin-and oxaliplatin-induced oxidative stress. In addition, curcumin has been shown to protect renal tissue from fibrosis by suppressing the transforming growth factor-β (TGF- β) in vitro [50].

Apart from renoprotective effects, a number of anti-dyslipidaemic activities of curcumin have been reported in diet- and/or chemical-induced dyslipidaemic animal models [51]. Furthermore, curcumin has shown significant potential in improving hepatic lipid metabolism in the non-CKD dyslipidaemic population [52, 53]. A recent study showed that curcumin ameliorates renal damage and oxidative stress in adenine-induced CKD in rats [54]. However, no study has examined the effects of curcumin on hepatic lipid alteration in experimentally induced CKD. Thus, the aim of the study was to examine the effects of increasing concentrations of curcumin on the various biochemical parameters involved in lipid metabolism of a chemically-induced CKD rat model to further support curcumin’s use in CKD.

## Methods

### Chemicals and apparatus used

Curcumin (> 94%) was purchased from Sigma (St. Louis, MO, USA). The diagnostic kits of cholesterol, triglycerides, HDL-cholesterol, albumin, creatinine and urea nitrogen were obtained from PM Separations (Capalaba DC, USA), while the non-esterified free fatty acid kit was obtained from Wako Diagnostics. Both the standard (AIN93G) and the adenine (0.75% w/w)-supplemented (SF15–082) rat pellet diets were supplied by Speciality Feeds (Glen Forrest, WA, Australia). The standard diet contained (in weight percentage) approximatley: 60% carbohydrate, 17.5% protein, 5% fat, 7% crude fibre, and the adenine-supplemented diet contained 0.75% adenine, in addition to the standard diet. All other chemicals were of analytical or higher grade from Sigma-Aldrich (St Louis, MO, USA), unless otherwise specified. A UV-VIS spectrophotometer (Ultrospec 2000, Biochrom Ltd., Cambridge, UK) was used for all absorbance measurements.

### Animals

In the present study, twenty-nine (29) adult male Sprague-Dawley rats with an average body weight of 150–200 g were used in polypropylene cages (3 rats per cage to minimise isolation stress) with water ad libitum and 12 h light/dark cycle in a temperature controlled facility at 24 ± 2 °C having 50–60% relative humidity. The rats were acclimatised to the laboratory conditions for one week prior to experimentation*.* The use and care of the animals in this experimental protocol was approved by the Institutional Animal Care and Ethics Committee (Approval Number: A11259) of Western Sydney University, Australia following the National Health and Medical Research Council (NHMRC) guidelines on the “Australian Code of Practice for the Care and Use of Animals for Scientific Purposes”.

### Experimental design and treatments

**Fig. 1:**
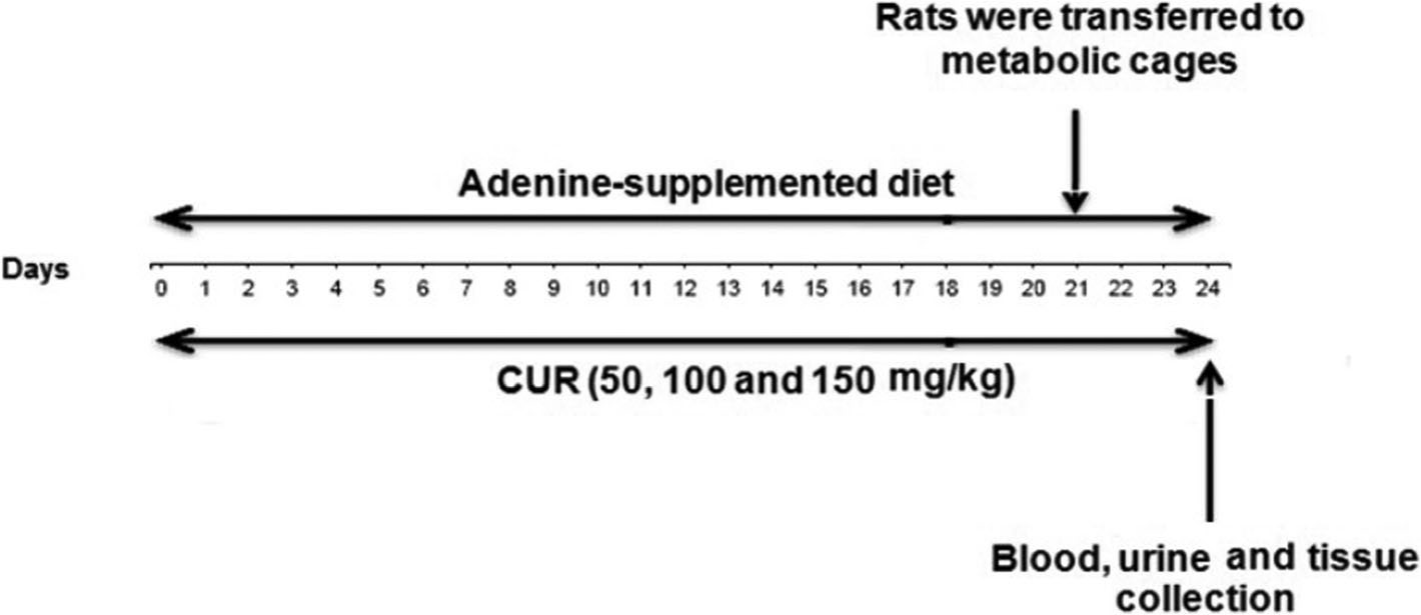
Schematic representation of experimental protocol

The sample size (number of rats per group) calculation in the present study was carried out using the “resource equation method” [55]. In this method, a value ‘E’ that indicates the sample size is measured which is the degrees of freedom of analysis of variance (ANOVA). The ‘E’ value is calculated by subtracting the total number of experimental groups from the total number of experimental animals. The rats were weight matched and randomly divided into five groups (*n* = 5 to 6 per group) and received the following treatments: Group 1 received 1% sodium carboxy methyl cellulose (CMC) together with standard diet and served as control; Group 2–5 received adenine-supplemented diet (0.75% w/w adenine in standard diet) to induce chronic kidney disease. In addition to adenine-supplemented diet, Group 2 received 1% sodium CMC and served as the CKD control; Group 3–5 received curcumin at doses of 50, 100 and 150 mg/kg (dissolved in 1% sodium CMC), respectively. All treatments were given by oral gavage once daily for 24 days. On day-21, the rats were placed individually in metabolic cages, acclimatised for two days and the 24 h faeces and urine were collected on day-24 (Fig. 1). The urine samples were centrifuged at 1000 rpm for 10 min to remove food particles and debris, and the supernatants were stored at − 20 °C until analysis. The rats were anesthetised with an intraperitoneal injection of ketamine (75 mg/kg) and xylazine (5 mg/kg) cocktail, and blood samples (approximately 3 mL) were collected from a cardiac puncture and allowed to clot for 30 min before centrifuging at 3000 rpm for 15 min. The serum was separated and stored at − 20 °C until biochemical analysis. After blood collection, the liver and kidney of each rat were immediately dissected, weighed and snap frozen in liquid nitrogen and stored at − 80 °C until biochemical analysis. At the end of the procedure, the rats were euthanised by exsanguination from the abdominal aorta.

## Biochemical estimations

### Body weight, food intake and water intake

The daily body weights of all rats were recorded daily at 4 PM for 24 days. The 24 h food and water intakes from each cage were determined daily at 10 AM.

### Determination of serum biochemical parameters

Serum total cholesterol, triglycerides, HDL-cholesterol, NEFA, urea nitrogen, albumin and creatinine were estimated as described [56] earlier using the commercial diagnostic kits following the manufacturer’s instructions. Serum VLDL and LDL-cholesterol were calculated indirectly by the Friedewald’s equations.

VLDL = Triglycerides/5; LDL = Total-cholesterol – [HDL + VLDL].

Atherogenic index (AI) and coronary risk index (CRI) as measures of the extent of atherosclerotic lesions and coronary atherosclerosis development, respectively, were calculated using serum total cholesterol and HDL cholesterol of different groups of rats using the mathematical formulae below [56].

AI = [Total cholesterol- HDL]/HDL; CRI = Total cholesterol/HDL.

### Determination of urine biochemical parameters

Total urinary protein (proteinuria) was estimated based on the method of Bradford following the manufacturer’s instructions (Bio-Rad, Hercules, CA, USA), with absorbance measured at 595 nm using Thermo Multiskan microplate reader. Urine creatinine and urea nitrogen (UUN) were estimated using the commercial diagnostic kits following the manufacturer’s instructions. Creatinine clearance was mathematically calculated as a clinical index of kidney function using serum and urine creatinine values as per the following formula [57].

[Urine creatinine (mg/dL) × urine volume (mL)/serum creatinine (mg/dL)] × [1000/body weight (g)] × [1/1440 (min)].

### Determination of hepatic lipids

Total lipids were extracted from the liver tissues by the modified method of Hara and Radin [58]. Briefly, 75–100 mg aliquots of liver tissue were homogenised in 20 volumes of isopropanol, shaken on orbital shaker for 45 min and centrifuged at 3000 x *g* for 15 min. The separated supernatants were analysed for hepatic total cholesterol, triglycerides and NEFA using commercial diagnostic kits.

### Data and statistical analysis

All the results are expressed as mean ± SEM. To analyse the quantitative differences among the experimental groups before or after treatments, the respective data was subjected to analysis of variance (ANOVA) using Graphpad Prism (version 6.0) statistical programme. Post-hoc comparisons were made using Dunnett’s multiple comparisons test. Statistical differences in individual groups before and after treatments were detected using Student’s paired t-test. In all tests, *p* < 0.05, *p* < 0.01 and *p* < 0.001 were used as the criterion criteria for statistical significance.

## Results

### Body weight

**Fig. 2:**
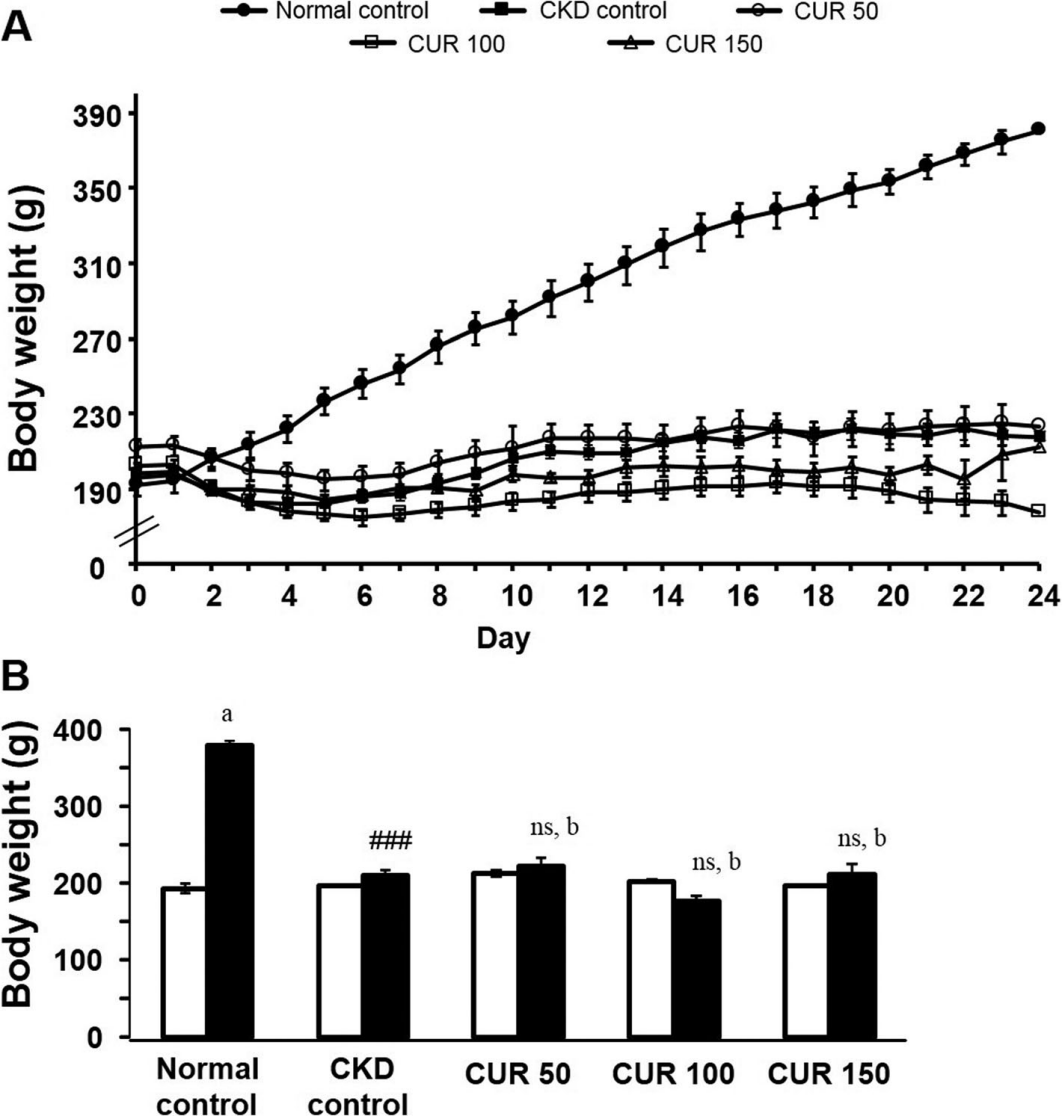
Effect of curcumin on adenine-induced body weight change in rats (A) Daily recordings of the mean body weight of the experimental groups of rats and (B) comparison of the mean body weights of rats prior to (open columns) and after treatment (closed columns) either with the adenine-supplemented diet alone or with curcumin. Each bar represents the mean ± SEM of 5–6 rats. Significant difference from normal control at identical times: ^###^ *p* < 0.001. No significant difference from CKD control rats at identical times: ns (*p* > 0.05). Significant difference from the respective pre-treated value: ^a^ p < 0.001. No significant difference from the respective pre-treated value: ^b^ (p > 0.05). CUR = Curcumin.

There was no significant difference in the initial body weights (194.2 ± 5.7 g to 198.8 ± 2.4 g; *n* = 5 to 6) among the different groups (Fig. 2 a). While the normal control rats (*n* = 5) exhibited a significant increase (380.4 ± 6.0 g vs 194.2 ± 5.7 g; *p* < 0.001) in body weight compared with their pre-treated values at the end of the 24 days, neither the CKD control nor the curcumin-treated rats showed any significant change in body weight compared with their pre-treated values. On the other hand, CKD control rats exhibited significant growth retardation (218.0 ± 5.2 g vs 380.4 ± 6.0 g; p < 0.001, *n* = 6) compared with the normal control group at the end of the 24 days (Fig. 2 b). However, the groups of rats treated with the various doses of curcumin (*n* = 6) did not show any signs of improvement in body weight compared with CKD control rats (Fig. 2 b).

### Water intake

**Fig. 3:**
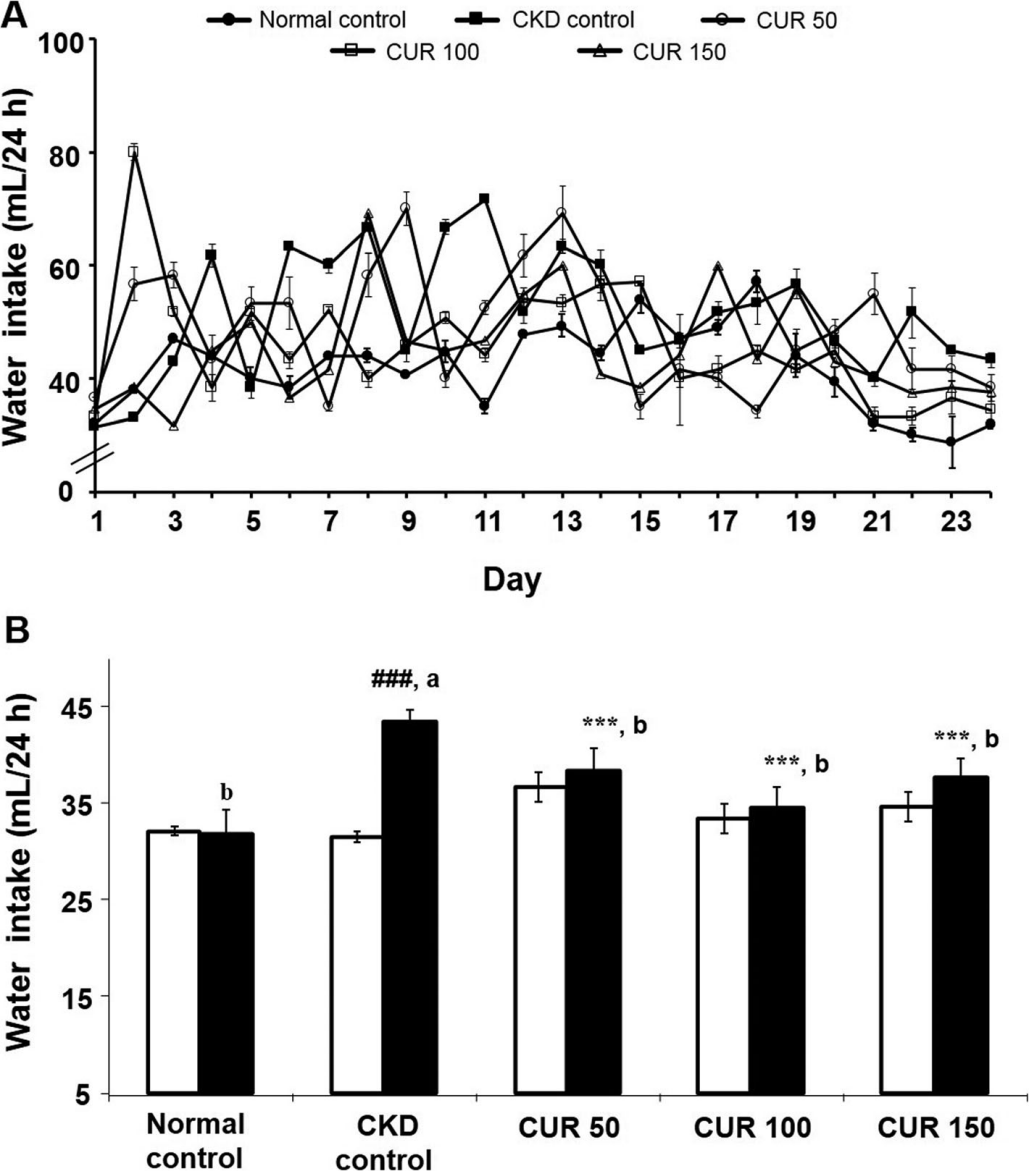
Effect of curcumin on adenine-induced water intake change in rats (A) Daily recordings of the mean water intake changes of the experimental groups of rats and (B) comparison of the mean water intake of rats prior to (open columns) and after treatment (closed columns) either with the adenine-supplemented diet alone or with curcumin. Each bar represents the mean ± SEM of 5–6 rats. Significant difference from normal control at identical times: ^###^ p < 0.001. Significant difference from CKD control rats at identical times: *** p < 0.001. Significant difference from the respective pre-treated value: ^a^ *p* < 0.001. No significant difference from the respective pre-treated value: ^b^ (p > 0.05). CUR = Curcumin.

There was no significant difference in the initial water intake (31.9 ± 1.1 mL to 31.6 ± 1.4 mL; *n* = 5 to 6) between different groups (Fig. 3 a). While the CKD control rats (*n* = 6) exhibited a significant increase in water intake compared with their pre-treated values (43.3 ± 1.1 mL vs 31.5 ± 1.2 mL; *p* < 0.001) or with the normal control group (43.3 ± 1.1 vs 31.9 ± 1.1 mL; *p* < 0.001) at the end of the 24 days, neither the normal control nor the curcumin-treated rats showed any significant change in water intake compared with their pre-treated values (Fig. 3 b). However, the groups of rats treated with the various doses of curcumin (*n* = 6) showed a significant decrease in water intake compared with CKD control rats (Fig. 3 b).

### Food intake

**Fig. 4:**
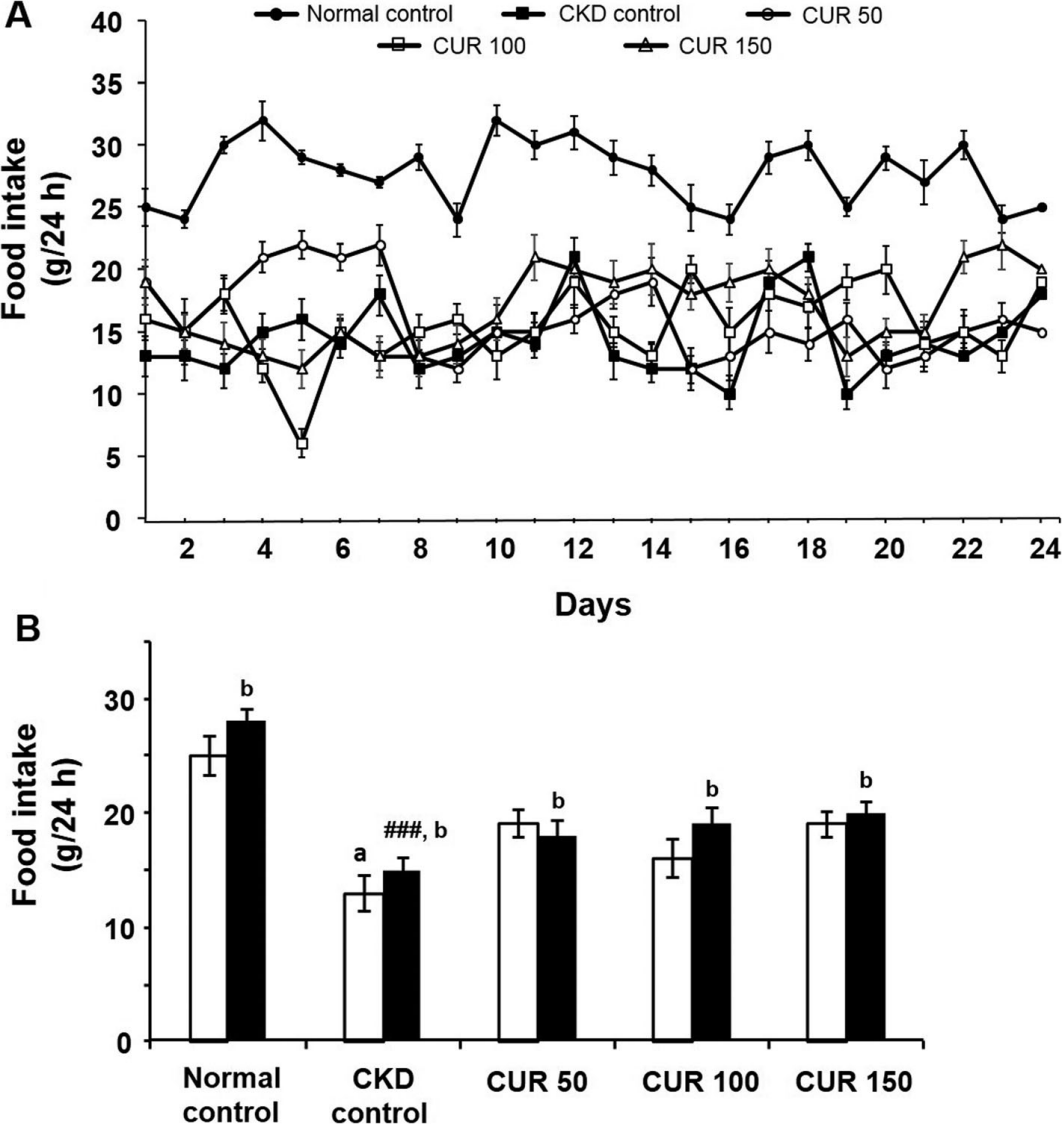
Effect of curcumin on adenine-induced food intake change in rats (A) Daily recordings of the mean food intake changes of the experimental groups of rats and (B) comparison of the mean food intake of rats prior to (open columns) and after treatment (closed columns) either with the adenine-supplemented diet alone or with curcumin. Each bar represents the mean ± SEM of 5–6 rats. Significant difference from normal control at identical times: ^###^ *p* < 0.001. Significant difference from normal control before the treatment: ^a^ *p* < 0.001. Significant difference from CKD control rats at identical times: *** *p* < 0.001. No significant difference from the respective pre-treated value: ^b^ (*p* > 0.05). CUR = Curcumin.

There was no significant difference in the initial food intake (19.3 ± 1.4 g to 13.9 ± 1.2 g; *n* = 6) between the CKD control and curcumin-treated groups (Fig. 4 a & b). However, there was a significant difference in the initial food intake (25.1 ± 1.7 g to 13.9 ± 1.2 g; *n* = 5 to 6) between the normal and the CKD control groups (Fig. 4 a & b). On the other hand, CKD control rats exhibited a significant (*p* < 0.001) decrease in food intake (28.3 ± 1.2 g vs 15.1 ± 1.1 g; n = 5–6) compared with the normal control group at the end of the 24 days (Fig. 4 b). However, the curcumin-treated groups (*n* = 6) did not show significant improvement in food intake compared with the CKD control group (Fig. 4 b).

### Serum total cholesterol

Rats fed with the adenine-supplemented diet (CKD control rats) alone showed a significant increase (*p* < 0.001, n = 6) in total cholesterol level when compared with the normal control group (*n* = 5; Table 1). Curcumin treatment produced a significant reduction in total cholesterol level at doses of 50 (*p* < 0.01, *n* = 6), 100 (*p* < 0.001, n = 6) and 150 mg/kg (*p* < 0.01, *n* = 6) compared with the CKD control rats, although the reduction was not dose-dependent among the three doses.

**Table 1:** Effect of curcumin treatment on serum lipid profile changes after 24 days in adenine-induced CKD rats

Parameter	Normal Control	CKD Control	CUR (50 mg/kg)	CUR (100 mg/kg)	CUR (150 mg/kg)
Total cholesterol (mg/dL)	82.11 ± 8.38	153.28 ± 4.64^###^	102.46 ± 11.09**	84.74 ± 3.91***	106.22 ± 13.52**
Triglycerides (mg/dL)	68.59 ± 3.82	109.19 ± 1.71^###^	85.42 ± 3.09*	65.65 ± 6.37***	75.70 ± 6.19**
HDL-cholesterol (mg/dL)	35.42 ± 3.10	16.16 ± 3.33^###^	23.44 ± 1.11^ns^	29.74 ± 1.11*	28.36 ± 3.41*
LDL-cholesterol (mg/dL)	32.97 ± 14.41	115.28 ± 5.23^###^	54.81 ± 5.35***	44.42 ± 4.28***	46.11 ± 4.09***
VLDL-cholesterol (mg/dL)	13.72 ± 0.76	21.84 ± 0.34^###^	17.08 ± 0.62*	13.13 ± 1.28***	15.14 ± 1.24**
NEFA (μEq/L)	872.63 ± 18.03	1099.16 ± 16.36^#^	810.92 ± 60.81^ns^	760.55 ± 37.07*	734.97 ± 78.61**
AI	1.31 ± 0.17	10.63 ± 2.28^###^	3.41 ± 0.48***	2.22 ± 0.58***	3.26 ± 1.17***
CRI	2.31 ± 0.17	11.63 ± 2.28^###^	4.41 ± 0.5***	3.22 ± 0.2***	3.10 ± 0.1****

Values represent the mean ± SEM of 5 to 6 rats

Significant difference from normal control: ^#^*p* < 0.05; ^###^p < 0.001

Significant difference from CKD control: ^*^p < 0.05; ^**^p < 0.01; ^***^p < 0.001

No significant difference from CKD control: ns (p > 0.05)

*CKD* Chronic kidney disease; *CUR* Curcumin; *NEFA* Non-esterified free fatty acids

*AI* Atherogenic index; *CRI* Coronary risk index

### Serum triglycerides

The CKD control group showed a significant (*p* < 0.001, *n* = 6) increase in serum triglycerides when compared with the normal control group (*n* = 5) at the end of the treatment (Table 1). Curcumin treatment at the doses of 50 (*p* < 0.05, *n* = 6), 100 (*p* < 0.001, *n* = 6) and 150 mg/kg (*p* < 0.01, *n* = 6) showed a statistically significant reduction in serum triglycerides compared to the CKD control group, although the reduction was not dose-dependent among the three doses.

### Serum HDL cholesterol

Rats fed with the adenine-supplemented diet alone showed a significant (*p* < 0.001; *n* = 6) reduction in HDL levels compared with the normal control (*n* = 5) fed with the standard diet at the end of the 24 days (Table 1). However, curcumin treatment at the doses of 100 and 150 mg/kg, along with adenine-supplemented diet, produced a significant (*p* < 0.05, *n* = 6) elevation in HDL cholesterol compared with the CKD control rats.

### Serum LDL and VLDL cholesterol

The LDL and VLDL levels were significantly elevated (*p* < 0.001; *n* = 6) in the CKD control rats as compared with the normal control rats (*n* = 5; Table 1). Curcumin treatment showed a significant reduction in LDL cholesterol at the doses of 50, 100 and 150 mg/kg (*p* < 0.001, *n* = 6) compared with the CKD control rats. On the other hand, curcumin treatment also showed a significant reduction in VLDL cholesterol at the doses of 50 (*p* < 0.05, *n* = 6), 100 (*p* < 0.001, *n* = 6) and 150 mg/kg (*p* < 0.01, *n* = 6) compared to the CKD control rats.

### Serum non-esterified fatty acids (NEFA)

Serum NEFA levels were found to be significantly (*p* < 0.05) higher in the CKD control group (*n* = 6) compared with the normal control rats (*n* = 5) at the end of the 24 days (Table 1). Curcumin treatment produced a significant reduction in serum NEFA at doses of 100 (*p* < 0.05, *n* = 6) and 150 mg/kg (*p* < 0.01, *n* = 6) compared with the CKD control rats.

### Atherogenic index (AI) and coronary risk index (CRI)

CKD control rats showed a significant (*p* < 0.001, *n* = 6) elevation of AI and CRI compared with normal control rats (*n* = 5; Table 1). On the other hand, the groups treated with curcumin at the three dose levels showed a significant (*p* < 0.001, *n* = 6) reduction in AI and CRI when compared with the CKD control rats.

## Kidney biochemical parameters

### Serum creatinine

The CKD control rats showed a significant increase (*p* < 0.001, *n* = 6) in serum creatinine level when compared with the normal control group (*n* = 5). Curcumin treatment produced a significant dose-dependent reduction (*p* < 0.01, *n* = 6) in serum creatine level at doses of 100 and 150 mg/kg (*p* < 0.01, *n* = 6) compared with the CKD control rats (Table 2).

**Table 2:** Effect of curcumin treatment for 24 days on serum and urine biomarkers of renal function in adenine-induced CKD rats

Biologic Fluid	Biomarker	Normal Control	CKD Control	CUR (50 mg/kg)	CUR (100 mg/kg)	CUR (150 mg/kg)
Serum						
	Creatinine (mg/dL)	0.45 ± 0.07	2.62 ± 0.08^###^	2.50 ± 0.15^ns^	1.71 ± 0.10**	1.27 ± 0.48**
	BUN (mg/dL)	4.46 ± 1.53	13.09 ± 1.19^##^	12.49 ± 2.53 ^ns^	7.14 ± 1.60*	4.99 ± 1.30**
	Albumin (mg/dL)	8.41 ± 0.32	5.47 ± 0.08^###^	6.57 ± 0.27 ^ns^	7.06 ± 0.39**	6.45 ± 0.27*
Urine						
	Urine volume (mL)	24.40 ± 2.21	37.00 ± 1.58^###^	32.50 ± 0.92	28.58 ± 1.46***	25.67 ± 1.17***
	Total protein (mg/24 h)	5.52 ± 0.83	70.73 ± 4.14^###^	25.26 ± 3.07***	14.82 ± 3.33***	18.13 ± 2.46***
	Creatinine (mg/dL)	28.69 ± 2.61	11.23 ± 0.34^###^	13.54 ± 0.85 ^ns^	22.72 ± 1.31***	23.56 ± 2.15***
	UUN (mg/dL)	14.28 ± 1.06	5.95 ± 0.75^###^	8.92 ± 0.82^ns^	9.96 ± 0.65**	10.01 ± 0.25**
	CrCl (mL/min/kg)	3.07 ± 0.20	0.63 ± 0.13^###^	0.53 ± 0.05 ^ns^	1.37 ± 0.15*	2.03 ± 0.23***

Values represent the mean ± SEM of 5 to 6 rats

Significant difference from normal control: ^##^*p* < 0.01; ^###^p < 0.001

Significant difference from CKD control: *p < 0.05; **p < 0.01; ***p < 0.001

No significant difference from CKD control: ns (p > 0.05)

*CKD* Chronic kidney disease; *CUR* Curcumin; *BUN* Blood urea nitrogen; *CrCl* Creatinine clearance; *UUN* Urine urea nitrogen

### Blood urea nitrogen

The blood nitrogen concentration (BUN) in the serum significantly increased (*p* < 0.001, *n* = 6) in CKD control rats compared with the normal control rats (*n* = 5). Curcumin treatment at the doses of 100 and 150 mg/kg significantly decreased (*p* < 0.05 and p < 0.01 respectively, *n* = 6) BUN concentration compared with the CKD control rats (Table 2).

### Serum albumin

The CKD control rats showed a significant (*p* < 0.001, *n* = 6) reduction of serum albumin level compared with the normal control rats (*n* = 5), which was significantly elevated by the curcumin treatment at the doses of 100 (*p* < 0.01, *n* = 6) and 150 (*p* < 0.05, *n* = 6) mg/kg at the end of the 24 day treatment period (Table 2).

### Urinary volume

The CKD control group showed a significant (*p* < 0.001, *n* = 6) increase in urinary volume when compared with the normal control group (*n* = 5) at the end of treatment. Curcumin treatment at the doses of 100 (*p* < 0.05, *n* = 6) and 150 mg/kg (*p* < 0.001, *n* = 6) showed a significant dose-dependent reduction in urinary volume compared to the CKD control group (Table 2).

### Urinary total protein (proteinuria)

The levels of total urinary protein in the CKD control rats were significantly increased (*p* < 0.001, *n* = 6) compared with the normal control rats (*n* = 5). However, all three doses of curcumin significantly reduced (*p* < 0.001, *n* = 6) proteinuria compared with the CKD control rats (Table 2).

### Urinary creatinine

The CKD control group showed a significant decrease (*p* < 0.001, *n* = 6) in urine creatinine when compared with the normal control group (*n* = 5) at the end of treatment. Curcumin treatment at the doses of 100 (*p* < 0.001, *n* = 6) and 150 mg/kg (*p* < 0.001, *n* = 6) showed statistically significant improvement in urine creatinine compared to the CKD control group (Table 2).

### Urinary urea nitrogen

The urinary urea nitrogen (UUN) concentration was significantly reduced (*p* < 0.001, *n* = 6) in the CKD control rats compared with the normal control rats (*n* = 5). Curcumin treatment at the doses of 100 and 150 mg/kg significantly increased (*p* < 0.05 and *p* < 0.01 respectively, *n* = 6) UUN concentration compared with the CKD control rats (Table 2).

### Creatinine clearance

The level of creatinine clearance calculated by the standard formula was significantly suppressed (*p* < 0.001, *n* = 6) in the CKD control rats compared with the normal control rats (*n* = 5; Table 2). However, curcumin treatment significantly improved creatinine clearance at the doses of 100 (*p* < 0.05, *n* = 6) and 150 mg/kg (*p* < 0.001, *n* = 6) compared with the CKD control rats.

### Hepatic lipid levels

There was no significant change in liver weight (normalised to body weight) observed among the experimental groups. Rats fed with adenine-supplemented diet alone (CKD control rats) showed a significant increase in hepatic cholesterol (*p* < 0.001, *n* = 6), triglycerides (*p* < 0.001, *n* = 6) and NEFA (*p* < 0.01, *n* = 6) compared with the normal control group (*n* = 5; Table 3). Curcumin at all the three doses significantly reduced the hepatic cholesterol (*p* < 0.05 to *p* < 0.001; *n* = 6), triglycerides (*p* < 0.01 to *p* < 0.001; *n* = 6) and NEFA (*p* < 0.05 to *p* < 0.001; *n* = 6) levels compared with the CKD control rats.

**Table 3:** Effect of curcumin treatment on liver weight and lipid levels after 24 days in adenine-induced CKD rats

Parameter	Normal Control	CKD Control	CUR (50 mg/kg)	CUR (100 mg/kg)	CUR (150 mg/kg)
Liver weight (g/100 g bw)	3.91 ± 0.09	3.61 ± 0.08	3.28 ± 0.23	3.01 ± 0.13	4.32 ± 0.50
Cholesterol (mg/g tissue)	0.38 ± 0.07	4.95 ± 1.30^###^	2.99 ± 0.28**	2.61 ± 0.15***	2.53 ± 0.24***
Triglycerides (mg/g tissue)	2.55 ± 0.73	10.51 ± 1.39^###^	5.00 ± 1.11**	2.65 ± 0.40***	3.73 ± 0.59***
NEFA (mg/g tissue)	0.73 ± 0.05	1.20 ± 0.06^##^	0.91 ± 0.11*	0.61 ± 0.07***	0.89 ± 0.05*

Values represent the mean ± SEM of 5 to 6 rats

Significant difference from normal control: ^##^*p* < 0.01; ^###^*p* < 0.001

Significant difference from CKD control: *p < 0.05; **p < 0.01; ***p < 0.001

*CKD* Chronic kidney disease; *CUR* Curcumin; *NEFA* Non-esterified free fatty acids;

## Discussion

In the present study, we examined the protective effects of curcumin on hepatic lipid derangement and renal damage in an adenine-induced rat model of CKD. The long-term feeding of adenine is known to suppress the excretion of various nitrogenous compounds due to renal tubular occlusion and produce metabolic abnormalities in rats that closely mimics CKD in humans [59]. Excess adenine in mammalian metabolism becomes a significant substrate for xanthine dehydrogenase and oxidises into low soluble compounds 2, 8-dihydroxyadenine which further precipitate in renal tubules and damage the kidney tissue accompanied by oxidative stress and subsequently renal dysfunction [60]. The adenine model is reproducible, simpler to conduct and more like human CKD than the 5/6 nephrectomy [61, 62]. Adenine-induced renal damage is time-dependent-the longer the feeding time, the more severe the renal damage [63]. Previous studies suggested that 0.75% w/w adenine in the diet for 4 weeks is an optimum duration for damage to the renal tissue to occur without mortality [61, 63]. Furthermore, adenine feeding for more than 7 weeks has caused significant mortality [64]. Nevertheless, Aoyama et al. [65] has shown that adenine (0.75% w/w) in the diet produced renal damage resembling CKD but also found 50% mortality. Thus, in the present study, we used adenine at the dose of 0.75% w/w in the diet for 24 days and no mortality was found which is consistent with previous reports [61–64]. Furthermore, biochemical evaluations in our study have shown that distinctive CKD was produced in rats upon feeding with adenine. To our knowledge, this is the first study to use an adenine-induced rat model of CKD to investigate the protective effects of curcumin on hepatic lipid metabolism.

In the present study, the body weight of the rats treated with curcumin was not significantly improved compared to CKD rats. The reason of the observed discrepancy in the body weight between curcumin-treated and untreated CKD rats is not clear. The possible explanation for this discrepancy is decreased food intake in CKD rats compared to control rats. Moreover, CKD resulted in impaired triglyceride rich lipoprotein (i.e. VLDL) metabolism which reduced the fatty acids delivery to the adipose tissue and limits the capacity to store energy and thus, contributes to weight loss, wasting and cachexia in CKD condition [66]. However, in the present study, curcumin could not reverse the CKD-induced weight reduction although it alleviated the impaired triglyceride metabolism in CKD rats. Furthermore, CKD rats increased water intake because the kidneys lose the capacity to concentrate the urine and excrete more water (which is evident by the increased urinary volume of CKD rats). Due to thirst, CKD rats drink more water. However, curcumin treatment normalised the water intake which may be due to its protective effects on kidney damage and thereby, the kidneys retain the capacity to concentrate the urine (which is evident by the decreased urinary volume of curcumin- treated rats).

CKD animals displayed a marked increase in serum total- and LDL-cholesterol. Serum total cholesterol elevation may be in part due to the enhanced cholesterol biosynthesis via up- regulation of the HMG-CoA reductase enzyme [67, 68] as well as due to a relative reduction of hepatic cholesterol elimination via down-regulation of the cholesterol 7α-hydroxylase (CYP7A1) enzyme in CKD animals [69, 70]. Moreover, increased serum LDL- cholesterol in the CKD rats could be due to the down-regulation of LDL receptor in response to CKD [71]. Although the precise molecular mechanism(s) of LDL-receptor protein deficiency in CKD have not been fully characterised, previous studies implicate inefficient translation and/or increased LDL-receptor protein turnover [72, 73]. However, our results demonstrated that chronic curcumin supplementation to CKD rats effectively reduced the serum and hepatic total-cholesterol and serum LDL-cholesterol, and this was found to be consistent with previous studies of curcumin using various dyslipidaemic animal models [74–76]. An earlier study demonstrated that curcumin reduced serum and hepatic cholesterol levels mainly by inhibiting HMG-CoA reductase enzyme in the liver [77–79]. It is reported that curcumin stimulates CYP7A1 enzymatic activity by increasing its hepatic gene expression, resulting in enhanced clearance of cholesterol as bile acids [80]. Moreover, previous studies demonstrated that curcumin up-regulates the expression of LDL receptor in mouse macrophages [81], human hepatoma derived HepG2 cells [82] and hepatic stellate cells (HSCs) [83]. Hence, the reduction of LDL-cholesterol by curcumin could be due to the prevention of the suppressive action of CKD on the LDL-receptor site. Thus, the constellation of these previous findings strongly support the results of our study and the observed cholesterol-lowering efffects of curcumin could be due to single or multiple effects of curcumin on potential site(s) of action leading to decreased cholesterol biosynthesis and/or enhanced elimination and/or increased hepatic uptake.

Hypertriglyceridaemia is one of the most common quantitative lipid abnormalities in patients with CKD [5, 84, 85]. Hypertriglyceridaemia in CKD is thought to be due to the dysregulation of various enzymes such as lipoprotein lipase (LPL) and hepatic lipase, apolipoproteins such as apoC III and apoC II and receptor such as VLDL-receptor involved in triglycerides metabolism [86]. In this study, CKD rats exhibited increased levels of serum and liver triglycerides (TGs) and serum TG-rich lipoprotein VLDL. Vaziri and Liang [87] reported that the deficiency of skeletal muscle, adipose tissue and myocardium LPL activities may contribute significantly to the elevation of TGs in CKD. Moreover, hepatic lipase protein expression and activity is also decreased in CKD rats [88, 89]. Collectively, lipase deficiency, at least in part, may be responsible for the apparent increased serum and hepatic triglyceride levels in CKD animals in the present study. The VLDL receptor belongs to the large LDL receptor gene family with distinctly different ligand specificity and tissue distribution compared with LDL receptor. It is primarily expressed in skeletal muscle, myocardium, and adipose tissue [90, 91] where it binds and internalises triglyceride-rich VLDL particle. It has been demonstrated that the down-regulation of VLDL receptor expression is associated with CKD and nephrotic syndrome together with elevated plasma VLDL levels [92]. Therefore, in this study, the increased serum VLDL levels in the CKD rats could be due to VLDL receptor deficiency.

Our results demonstrated that chronic curcumin supplementation effectively reduced serum triglycerides and VLDL-cholesterol along with liver triglycerides in accordance with earlier reports [79, 93, 94]. Previously, Seo et al. [95] demonstrated that chronic curcumin supplementation increased skeletal muscle LPL activity in *db/db* mice. More recently, Prabu and Sumedha [96] reported that a curcumin analogue increased plasma LPL activity in arsenic intoxicated rats. Thus, in the present study, the reduction in triglycerides levels following curcumin administration could be due to increased tissue and/or plasma LPL activity.

Together with hypertriglyceridaemia, CKD is associated with impaired hepatic fatty acid metabolism. Consistent with previous reports, CKD rats exhibited a significant elevation of serum and hepatic free fatty acids in our study. Chronic curcumin administration markedly reduced the serum and hepatic free fatty acid levels. Although little is known on the effects of CKD on fatty acid metabolism, it has been demonstrated that curcumin decreased lipid accumulation by up-regulating PPARα while down-regulating various lipogenic genes such as SREBP1c, acetyl-CoA carboxylase 1 (ACC1), fatty acid synthase in the liver of mice [97, 98]. Thus, curcumin may regulate hepatic fatty acid metaboilsm in CKD by decreasing synthesis and/or increasing catabolism in liver.

The ratio of total cholesterol to HDL serves as a useful indicator of cholesterol homeostasis in the arterial wall, glomerular mesangium and liver. The CKD model employed in the present study exhibited a significant increase in serum total cholesterol-to-HDL cholesterol ratio (CRI), indicating an atherogenic profile. In contrast, chronic curcumin supplementation to CKD animals normalised total cholesterol-to-HDL cholesterol ratio (CRI) and AI. Furthermore, CKD is consistently associated with reduced plasma HDL cholesterol level largely due to the impaired maturation of cholesterol ester poor HDL-3 to cholesterol rich HDL-2. In the present study, CKD rats demonstrated a decreased concentration of HDL compared to control rats. HDL abnormalities in CKD are largely due to lecithin–cholesteryl acyltransferase (LCAT), an important enzyme for HDL maturation, deficiency [67] and/or reduced expression of hepatic HDL docking receptor (SR-B1) [99–101]. ATP-binding cassette transporter A1 (ABCA1) is a membrane associated protein that mediates transfer of cellular cholesterol and phospholipids to lipid-poor HDL for disposal in the liver and, as such, serves as a gatekeeper of reverse cholesterol transport pathways [102]. Prabu and Sumedha [96] reported that arsenic-intoxicated rats chronically treated with dimethoxycurcumin, a structural analogue of curcumin, showed increased LCAT activity which also supports an earlier observation by Tu et al. [103] in high-fat diet-fed rats. Moreover, Zhao et al. [104] demonstrated that curcumin dose-dependently increased the protein level of ABCA1 in mouse macrophages but did not affect the protein expression of SR-BI. Thus, in the present study, the molecular mechanism(s) of curcumin to alleviate the HDL abnormalities could be due to the inhibition of the suppressive action of CKD on LCAT and/or ABCA1 protein deficiency.

Moreover, in the present study, curcumin failed to show dose-dependence on lipid parameters measured in plasma and in the liver and kidney tissues. The values indicate that the high dose (150 mg/kg) showed more or less similar size effect (statistically non-significant difference) as that of the median dose (100 mg/kg). As pointed out by previous researchers, curcumin is poorly soluble in aqueous solutions and hence, its maximum solubility in gastric fluids would have been reached with around 100 mg/kg dose in our study. Thus, one possible explanation for curcumin’s failure to show dose-dependent effects could be due to its poor aqueous solubility in gastric fluids leading to inadequate intestinal absorption and low oral bioavailability [105–107].

Limitations of the present study include evaluating the protective effects of curcumin only and does not provide any information on the curative effects of curcumin on CKD-induced dyslipidaemia. Furthermore, we have deduced the molecular mechanism(s) responsible for the observed changes in lipid metabolism from literature. Further studies have been undertaken in our laboratory to explain the mechanism(s) of the hepatic lipid metabolism-regulating activities of curcumin (manuscript in progress).

## Conclusion

CKD induced by feeding adenine-supplemented diet to rats leads to the development of metabolic dyslipidaemia. Long-term curcumin administration improves metabolic dyslipidaemia and shows renoprotective effects in adenine-induced CKD. Thus, the present findings support the potential therapeutic value of curcumin as a protective phytoconstituent in attenuating CKD-induced cardiovascular disease, although further clinical evaluation is required as a treatment modality.

## Data Availability

The datasets used and/or analysed during the current study are available from the corresponding author on reasonable request.

## Abbreviations

ACAT: Acyl
CKD: Chronic kidney disease
CoA: Cholesterol acyltransferase
CRF: Chronic renal failure
CVD: Cardiovascular disease
CYP7A1: Cholesterol 7α-hydroxylase
ESRD: End stage renal disease
HDL: High-density lipoprotein
HMGCoA: 3-hydroxy-3-methyl-glutaryl-coenzyme A
LCAT: Lecithin–cholesterol acyltransferase
LDL: Low-density lipoprotein
LDLr: Low-density lipoprotein receptor
LPL: Lipoprotein lipase
NEFA: Non-esterified free fatty acid
PCSK9: Proprotein convertase subtilizing kexin 9
SR-B1: Scavenger receptor class B type 1
STZ: Streptozotocin
TG: Triglycerides
VLDL: Very low-density lipoprotein
